# Influence of Different Modifiers on Bonding Strength and Rheological Performance of Bitumen Emulsion

**DOI:** 10.3390/ma12152414

**Published:** 2019-07-29

**Authors:** Changluan Pan, Deqiang Liang, Liantong Mo, Martin Riara, Juntao Lin

**Affiliations:** 1 State Key Lab of Silicate Materials for Architectures, Wuhan University of Technology, Wuhan 430070, China; 2 China Gezhouba Group No.5 Engineering Co., Ltd., Yichang 4430002, China; 3 Department of Physical Sciences, South Eastern Kenya University, P.O. Box 170-90200, Kitui 90200, Kenya; 4 Faculty of Engineering, China University of Geosciences (Wuhan), Wuhan 430074, China

**Keywords:** bitumen emulsion, emulsion modification, bonding strength, rheological property

## Abstract

Styrene butadiene rubber latex (SBR), waterborne epoxy adhesive (WE) and colloidal silica sol (SiO_2_) were used to prepare modified bitumen emulsion for cold mix asphalt. The modification effects of the individual modifiers and the combination of these modifiers were investigated by using bonding strength and dynamic shear rheological property. Test results showed that the modifier dosage helped to balance the performance of modified bitumen emulsion by improving its bonding strength without compromising its rheological properties. The critical dosage at which the peak bonding strength occurred was 4%, 12% and 4% for SBR, WE and SiO_2_ respectively. Improved rheological performance on the master curves was well distinguished, in particular, by increased complex modulus and reduced phase angle at the low frequency region. Abrupt changes, especially on phase angle occurred when the modifier dosage was beyond 12%. The measured ratio between bonding strength and complex shear modulus could vary ranging from 10^−2^ to 10^2^. Highly-modified bitumen emulsion with good adhesion, rheology and compatibility can be prepared by using the combination of SBR, WE and SiO_2_. It is important to carefully select the type and dosage of modifier for a particular combination to optimize the performance of modified bitumen emulsion.

## 1. Introduction

Cold mix asphalt mixtures prepared using bitumen emulsions, unheated aggregates and filler, are considered as a viable alternative to make asphalt pavements more economic, less polluting, and with lower energy consumption. Cold recycling asphalt mixture could be prepared with high contents of reclaimed asphalt pavement (RAP), hence reducing the consumption of virgin materials and lowering energy needs [1,2]. Compared with traditional hot mix asphalt, cold mix asphalt, especially containing RAP, is a more sustainable material for pavement construction due to its long term benefits to the environment [3,4]. Nevertheless, cold mix asphalt has several limitations including low initial strength, long curing time, excessive permanent deformation, weak adhesiveness, susceptible thermal cracking and low durability [5]. For this reason, cold mix asphalt is commonly used as a base course for high-quality pavements or a surface course for secondary roads with a protective wearing surface [6]. For instance, micro surfacing, which is a mixture of polymer-modified bitumen emulsion, quality aggregates, filler and water, is commonly applied as a protective wearing surface course for the purpose of pavement preservation [7]. This indicates that cold mix asphalt with high quality and performance has the potential for application in various courses of asphalt pavement systems. 

Several studies have been done to improve the performance of cold mix asphalt by using various additives. Effects of additives on the mechanical performance in recycled mixtures with bitumen emulsion can be found in [8]. Active fillers including cement and lime are the commonly used additives instead of conventional stone fillers [9,10]. Furthermore, polymer modified bitumen emulsion is another effective solution, which can provide many advantages including enhanced setting, higher early strength, improved cohesion and better adhesion [11,12]. The combination of active filler and polymer-modified bitumen emulsion usually facilitates a more effective improvement on the performance of cold mix asphalt. 

Chemical additives were recently used to further improve the performance of cold recycling asphalt mixtures. El-Rahman evaluated the performance of bitumen emulsion modified using epoxy resin, latex styrene butadiene styrene (SBS), poly vinyl acetate (PVA), and with a mixture of two or more types of the aforementioned polymers. Bitumen emulsion modified with a mixture of epoxy resin, latex SBR and PVA showed the best road performance in terms of fuel and acid resistance. The dynamic viscosity test on the residues indicated a promising high viscosity value for different pavement applications [13]. Zhang evaluated the performance of a tack coat bitumen emulsion modified using waterborne epoxy resin blended with styrene-butadiene rubber (SBR). Addition of SBR latex and epoxy improved the high and low temperature properties as well as shear strength significantly [14]. Hu performed a laboratory evaluation of waterborne epoxy bitumen emulsion designed for pavement preventative maintenance application. When the bitumen emulsion modified by 50% waterborne epoxy was used to coat 0.45–0.9 mm aggregates, it formed a durable coating layer and the skid resistance of the aggregates was improved significantly [15]. Ji studied the performance of cold mix asphalt prepared by using waterborne epoxy resin-modified bitumen emulsion. Compared with hot asphalt mix, a significant increase in rutting resistance at high temperatures and fair moisture resistance was observed, while the low temperature and fatigue performance properties were reduced. Micro surfacing prepared with waterborne epoxy resin-modified bitumen emulsion also indicated a promising performance on resistance to moisture damage, skid and rutting [16,17]. Test results on nano-silica-modified bitumen emulsion indicated that nano SiO_2_ reduced the ductility and penetration of the bitumen emulsion residue [18]. Chen reported that bitumen emulsion modified with 3% SBR and 0.05% nano-silica had improved high temperature properties. The improvement was attributed to the reticular construction of the nano-silica/SBR composite and the swelling of the SBR after oil absorption in the system [19]. Sheng et al indicated that modified emulsified asphalt containing 6% polyurethane showed good improvement on penetration, softening point and ductility. The improvement effects on penetration and ductility was better than that of SBS, but both modifiers had similar effects on softening point [20]. Bitumen emulsion was modified by nanoscale polyurethane emulsion by means of gentle agitation method. Dynamic rheological test showed increased complex modules and reduced phase angle, which indicated that the resistance to permanent deformation was enhanced [21]. In a similar study, Carrera assessed the properties of polyurethane-modified bitumen emulsions for cold mix applications. Addition of 4 wt% polyurethane resulted in a modified emulsion residue with a better resistance to permanent deformation when compared with neat bitumen [22].

Previous studies indicated that use of modified bitumen emulsion improves the performance of cold mix asphalt. Various modifiers have been used to modify bitumen emulsion in order to improve its performance properties. In this study, SBR latex, waterborne epoxy adhesive and colloidal silica sol were used to prepare modified bitumen emulsion. The main aim was to get an insight into the modification effects of individual modifier and the combination of two or three of the selected modifiers. Bonding strength test and dynamic shear rheological test were carried out on the residues of modified bitumen emulsion. Improvement on bonding strength and rheological properties was used to determine the optimal dosage of each modifier. Bitumen emulsion modified with the combination of SBR latex, waterborne epoxy adhesive and colloidal silica sol was also prepared. The combined modification effects were examined in order to assess whether improved rheological properties could be obtained without compromising the bonding strength. Finally, the feasibility of preparing highly-modified bitumen emulsion using blended modifiers was analyzed.

## 2. Materials and Methods

### 2.1. Materials

The bitumen emulsion used in this study is a commercial cationic slow set bitumen emulsion for cold mix applications. It met the related technical requirements on BC-1 base bitumen emulsion according to Technical Specification for Construction of Highway Asphalt Pavements (JTG F40-2017) [23]. The binder content was 52% and the emulsion residue had a penetration of 5.2 mm, softening point of 47 °C and ductility larger than 100 cm, respectively. Bitumen emulsion was modified using SBR latex, waterborne epoxy adhesive and colloidal silica sol (SiO_2_). SBR latex was a milky white liquid with a solid content of 55% and a viscosity of 343 MPa·s. The waterborne epoxy adhesive consisted of two components including a waterborne epoxy resin and a waterborne curing agent. The waterborne epoxy resin is an emulsified epoxy with a mean particle size of 0.5 µm, solid content of 52% and epoxy equivalent on solids of 510 g/Eq. The epoxy curing agent was a yellow viscous liquid with an amine value of 186 mg KOH/g and a rotary viscosity of 6200 mPa·s. The waterborne epoxy adhesive was prepared by blending the waterborne epoxy resin and curing agent at a mass ratio of 1:1. The colloidal silica sol was an acidic non-stabilizer type with a SiO_2_ content of 30%, average particle diameter of about 10 nm and pH value of 3. 

Neat bitumen 70# (Penetration 60–80) and Styrene-Butadiene-Styrene (SBS) polymer-modified bitumen were used for the purpose of comparison with the emulsion residues in terms of dynamic shear rheological properties. The neat bitumen had a penetration of 7.4 mm, softening point of 47 °C and ductility higher than 100 cm at 15 °C. The SBS-modified bitumen had a penetration of 4.7 mm, softening point of 65 °C and ductility higher than 100 cm at 15 °C. SBS-modified bitumen is commonly used in the construction of the surface wearing course and intermediate layer, while neat bitumen is used in the bottom layer of most three-layer asphalt pavements in northern China. 

### 2.2. Preparation of Modified Bitumen Emulsion

In this study, modified bitumen emulsion was prepared by adding modifier into bitumen emulsion and shear blending at a speed of 300 rph for 5–10 min. Table 1 gives a summary of modifier dosages for various types of modified bitumen emulsion. For bitumen emulsion containing combined modifiers, SBR modifier was first added into bitumen emulsion and blended to obtain well dispersed emulsion. Waterborne epoxy adhesive was then added and blended after which SiO_2_ was added and blended. It should be noted that waterborne epoxy resin and waterborne curing agent should be well mixed before adding it into bitumen emulsion. The residue of modified emulsified asphalt was recovered by coating bitumen emulsion on a glass plate with a thin film of around 2 mm and letting it dry naturally. 

### 2.3. Bonding Strength Testing

**Figure 1:**
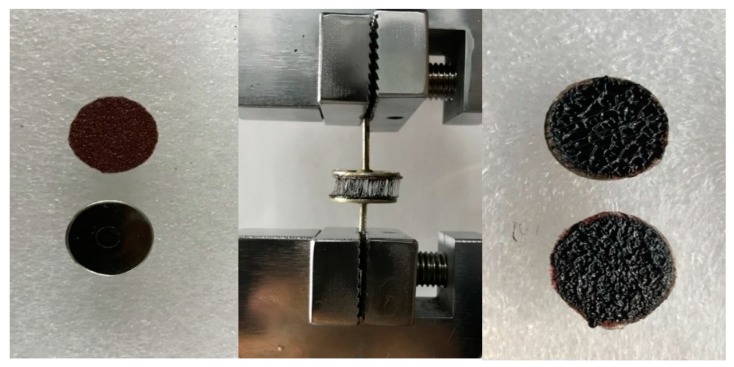
Flat head steel tacks (**left**), test setup (**middle**) and the fractured surfaces after testing (**right**).

The test sample for bonding strength of bitumen emulsion was prepared by using a pair of flat head steel tacks with a diameter of 14 mm as indicated by Figure 1. One of the steel tacks was glued with a sandpaper (mesh 60) to facilitate emulsion coating. Three drops of bitumen emulsion were applied on the sandpaper surface and allowed to dry at room temperature for 24 h in order to form a residue film of about 1 mm thickness. A thin film of liquid glue was applied on another flat head tack. Later, both steel tacks were adhered together and allowed to bond for 3 h, after which the samples were preconditioned at the test temperature for 1 h. Three test temperatures were considered including −10 °C, 20 °C and 60 °C. A tensile testing machine (ZQ-990A, Zhiqu Precision Instruments Co., Ltd., Dongguan, China) was used to carry out the bonding strength test at a loading rate of 50 mm/min. The bonding strength of different modified bitumen emulsion was calculated as the maximum force divided by the cross-sectional area of test samples.

### 2.4. Dynamic Shear Rheological Test

A dynamic shear rheometer (DSR) (MCR-101, Germany) was used to characterize the rheological properties. A frequency sweep test was performed at different temperatures ranging from −10 °C to 60 °C with an interval of 10 °C. For each test temperature, the test frequency ranged from 0.1 to 400 rad/s. When the test temperature was below 20 °C, the 8 mm diameter parallel plates and 2 mm gap were used and, when the test temperature was above 20 °C, 25 mm diameter parallel plates and 1 mm gap were used. From the frequency sweep test, the complex modulus (G*) and phase angle (δ) were obtained. By means of the time temperature superposition principle (TTSP), the δ and G* data at different temperatures were horizontally shifted to the reference temperature of 20 °C to construct the rheological master curves. 

## 3. Results and Discussion

### 3.1. Bonding Strength Test Results

**Figure 2:**
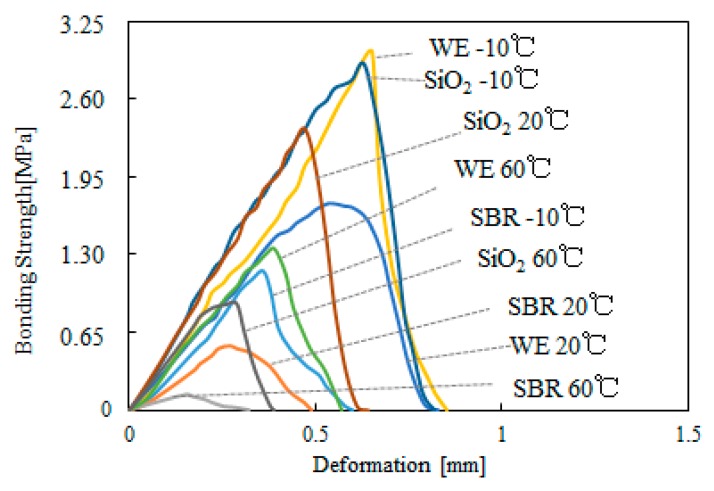
Typical strength–displacement curves of bonding strength tests.

Figure 2 presents an example of some strength–displacement curves obtained from bonding strength tests done on different types of modified bitumen emulsion. In this figure, only three types of bitumen emulsions with 4% of SBR, 12% of WE, and 4% of SiO_2_ was specially selected to well distinguish the curves from each other. For the sake of brevity, the rest curves are not discussed. The test was conducted at −10 °C, 20 °C and 60 °C. Figure 2 shows that the effects of temperature and type of modifier is distinct. In general, increasing temperature resulted in lower bonding strength. The effect of type of modifier was more complex and depended on test temperature. For example, at a temperature of 20 °C, SiO_2_-modified bitumen emulsion showed the maximum bonding strength followed by WE-modified bitumen emulsion, and finally SBR-modified bitumen emulsion. At −10 °C and 60 °C, WE-modified bitumen emulsion had the highest binding strength, followed by SiO_2_-modified bitumen emulsion, and finally SBR-modified bitumen emulsion. All of the fracture surfaces after bonding strength tests were examined. It was found that both of the fracture surfaces of each sample were covered by bitumen film independent of test temperature. This indicated a cohesive failure of bitumen film. Adhesive failure at the sandpaper or the steel tacks surfaces was not observed. As test temperature increased, bitumen became softer and resulted in lower cohesive strength. For this reason, bitumen tested at 20 °C and 60 °C showed a cohesive failure regardless of the type of modifier. 

**Figure 3:**
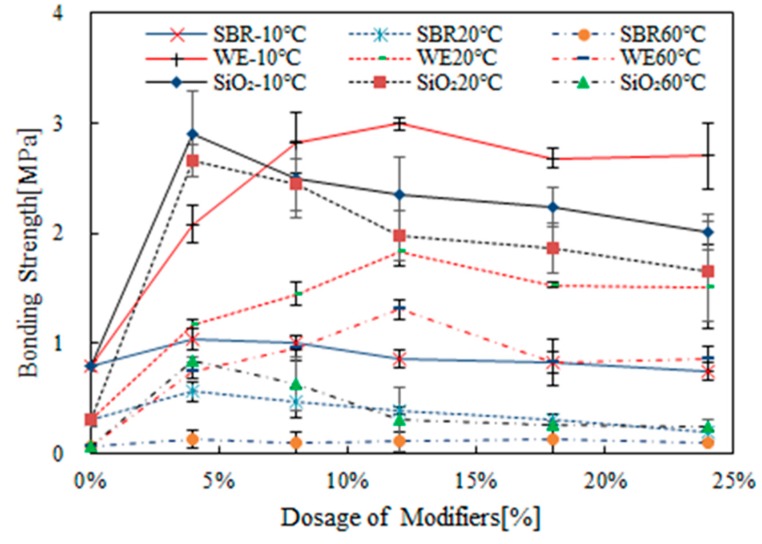
Effect of modifier dosage and temperature on bonding strength of various types of modified bitumen emulsion.

Figure 3 shows the effect of modifier dosage and temperature on bond strength of various types of modified bitumen emulsion. The modifier dosage range from 0 to 24% and test temperature was −10 °C, 20 °C and 60 °C. For SBR-modified bitumen emulsion, the bonding strength increased at the beginning and reached a peak value at 4% SBR dosage. Further increase in SBR dosage led to a reduced bonding strength. For example, bitumen emulsion with 4% SBR dosage had the highest bond strength of 0.56 MPa at 20 °C. A peak value of 1.05 MPa was also observed at −10 °C. At a higher temperature of 60 °C, the effect of SBR dosage was relatively small.

As observed in Figure 3, WE modification significantly improved the bonding strength of bitumen emulsion. With increase in WE dosage, the bonding strength increased gradually. When the WE dosage reached 12%, the bonding strength exhibited a maximum value. Further increasing the amount of WE led to a steady reduction in bonding strength. WE-modified bitumen emulsion showed less temperature sensitivity but larger bonding strength, especially at 60 °C when compared with SBR-modified bitumen emulsion. The bonding strength of WE-modified bitumen emulsion was significantly higher than that of base bitumen emulsion, especially at higher temperatures.

As shown in Figure 3, the addition of SiO_2_ improved the bonding strength significantly. When SiO_2_ dosage reached 4%, the bond strength had a maximum value regardless of test temperature. When SiO_2_ dosage was beyond 4%, the bonding strength decreased continually. The effect of temperature on bonding strength was obvious at a high temperature. Only a slight difference was observed for samples tested at 20 °C and −10 °C. 

**Figure 4:**
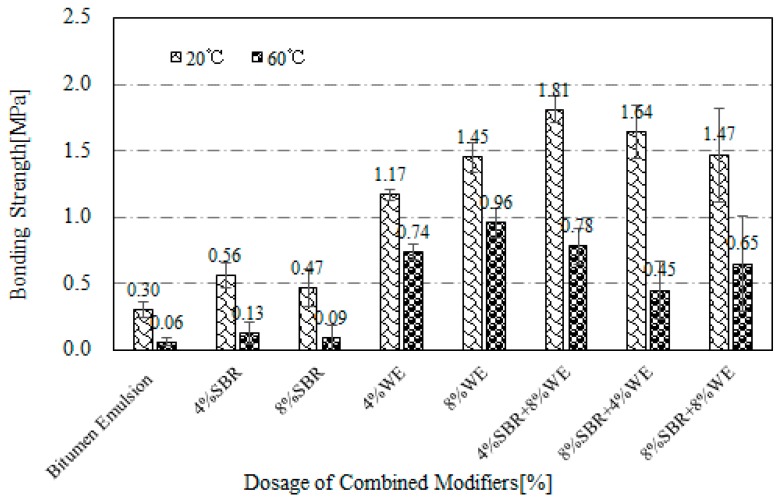
Bonding strength of Styrene butadiene rubber latex (SBR) and waterborne epoxy adhesive (WE)-modified bitumen emulsion.

**Figure 5:**
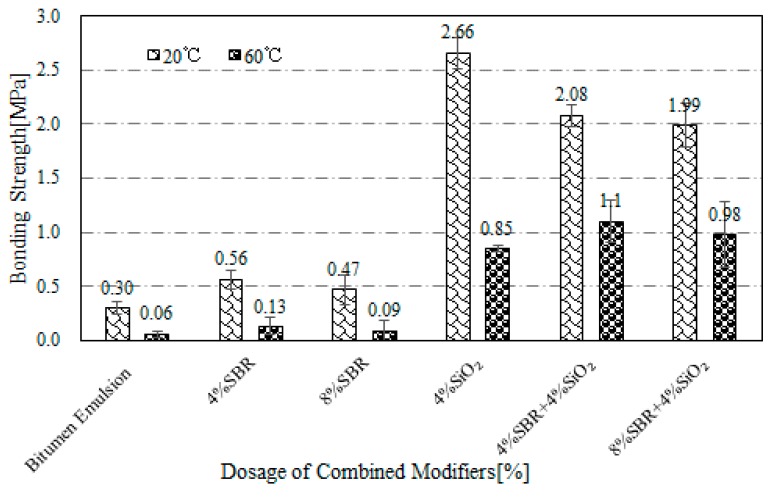
Bonding strength of SBR + SiO_2_-modified bitumen emulsion.

**Figure 6:**
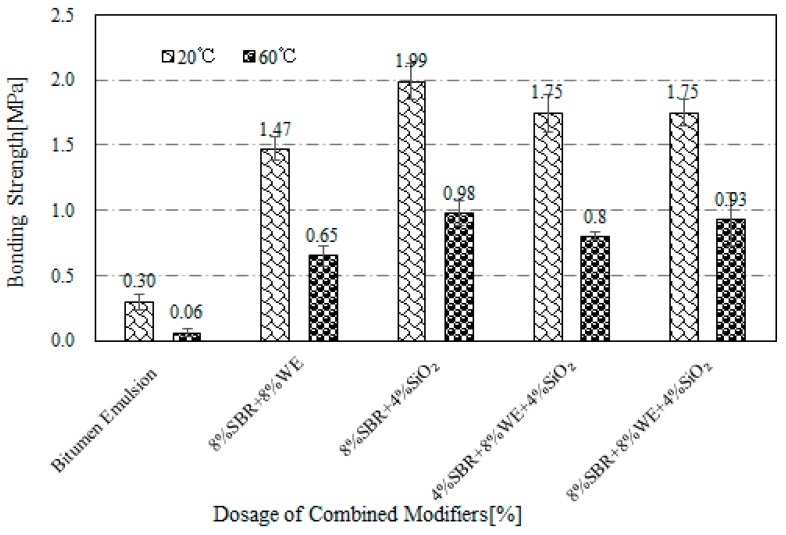
Bonding strength of SBR + WE + SiO_2_-modified bitumen emulsion.

In general, the optimum dosage range of the individual modification could be determined by the dosage at which the peak value of bonding strength appeared. As indicated in Figure 3, one can determine that the optimum dosage of SBR, WE and SiO_2_ as 4%, 12% and 4% respectively. In order to investigate the effects of combined modification between SBR + WE, SBR + SiO_2_ and SBR + WE + SiO_2_ on the bonding properties, bitumen emulsion containing different modifier combinations at various dosage were prepared and tested. Figure 4, Figure 5 and Figure 6 show the bonding strength results for bitumen emulsion after combined modification.

As indicated in Figure 4, bitumen emulsion modified with SBR + WE had an improved bonding strength compared with the emulsion modified separately with SBR and WE. Among the three SBR + WE combinations, 4% SBR + 8% WE showed the highest bonding strength. This indicated that the combination of low dosage of SBR and high dosage of WE could be promising. 

Figure 5 presents the results of bonding strength of SBR + SiO_2_-modified bitumen emulsion at 20 °C and 60 °C. The bonding strength of SBR + SiO_2_-modified bitumen emulsion was greatly improved compared with unmodified emulsion. The bonding strength of bitumen emulsion under combined modification with 4% SBR + 4% SiO_2_ at 20 °C was 2.08 MPa, while the bond strength of the same emulsion under single modification with 4% SBR and 4% SiO_2_ was 0.56 MPa and 2.66 MPa respectively. This indicated that the addition of SiO_2_ into SBR-modified bitumen emulsion can further improve the bonding strength.

Figure 6 shows the test results for bonding strength of SBR + WE + SiO_2_-modified bitumen emulsion. There was a small difference between the bonding strength of 4% SBR + 8% WE + 4% SiO_2_ and 8% SBR + 8% WE + 4% SiO_2_. The bond strength of the three-modifier combination was higher than that of SBR and WE single modification, but less than that of SiO_2_ single modification. High bonding strength also indicated a good compatibility of the three modifiers in bitumen emulsion. It also implied that SiO_2_ had the most significant effect on bonding strength and thus could be used as an adhesive promotor in the system of blended modifiers.

### 3.2. Dynamic Shear Rheological Analysis

DSR rheological analysis was conducted on the residue of various types of modified bitumen emulsion. Frequency sweep testing was carried out at different temperatures. In order to construct the master curves of complex modulus and phase angle, a reference temperature of 20 °C was selected and test data obtained at other test temperatures were shifted horizontally to overlap with that of the reference temperature. This yielded a smooth curve based on time temperature superposition principle (TTSP). In this way, the complex modulus and phase angle at high temperature were translated to the low frequency region, while those at low temperatures were translated to the high frequency region.

**Figure 7:**
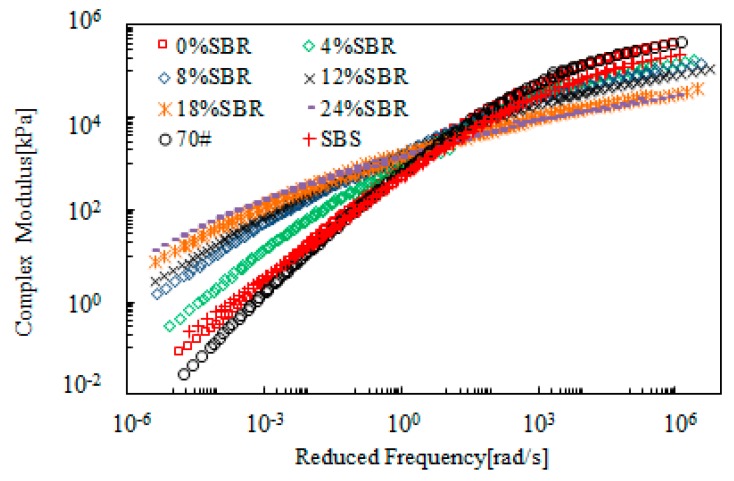
Complex modulus master curves of SBR-modified emulsified asphalt.

Figure 7 shows the master curves of complex modulus obtained from SBR-modified bitumen emulsion. The SBR dosage ranged from 0 to 24%. For the purpose of comparison, neat bitumen (70#) and SBS polymer-modified bitumen was also tested and the data obtained is also included in this figure. Figure 7 shows that addition of SBR resulted in lower complex modulus at the high reduced frequency range, and a contrary trend at the low reduced frequency range. This indicated an improvement on both low- and high- temperature properties. When SBR dosage was beyond 4%, the modified bitumen emulsion exhibited better rheological properties compared to SBS-modified bitumen. Further increase in SBR dosage improved the rheological properties. This is desirable for resistance to both low-temperature cracking and high-temperature rutting.

**Figure 8:**
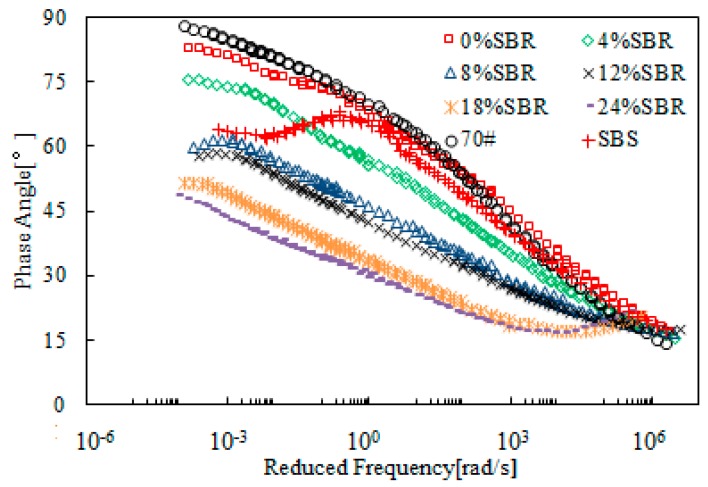
Master curves of phase angle of SBR-modified bitumen emulsion.

Figure 8 presents the master curves of phase angle of SBR-modified bitumen emulsion. Addition of SBR reduced the phase angle over the whole range of reduced frequency when compared with 70# neat bitumen. SBS-modified bitumen showed a plateau, which indicated the strong modification effect of SBS polymer. However, such a plateau did not exist for SBR-modified bitumen emulsion. When SBR dosage was 8%, the master curve of modified bitumen emulsion was located below the one for SBS-modified bitumen with a phase angle lower than 60°. Further increase in SBR dosage resulted in a lower phase angle. In general, the results of complex modulus and phase angle indicated that 8% SBR dosage was a critical dosage for modified bitumen emulsion.

**Figure 9:**
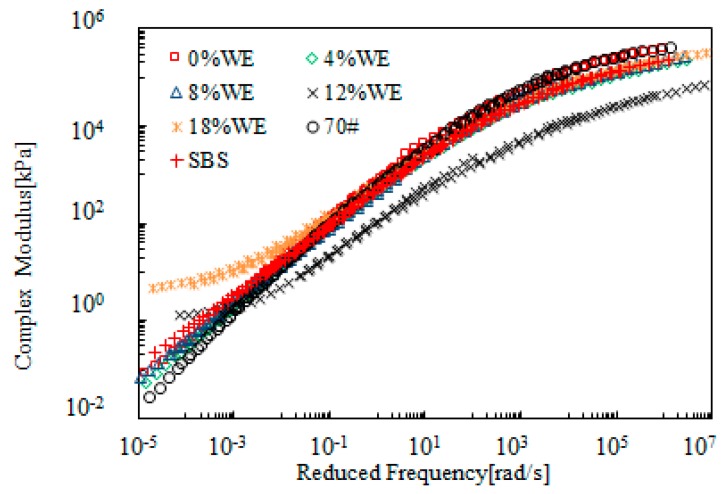
Master curves of the complex modulus of WE-modified bitumen emulsion.

Figure 9 shows the master curves of the complex modulus of WE-modified bitumen emulsion. Addition of WE increased the complex modulus at low frequency region, which corresponds to high temperature. The complex modulus of 12% WE-modified bitumen emulsion was larger than that of SBS-modified asphalt. The residue of 24% WE-modified bitumen emulsion became hard and brittle and thus could not be tested. The reduction of complex modulus at high frequency area was also observed with the addition of WE. The complex modulus of 12% WE-modified bitumen emulsion reached a minimum value at the high frequency region. 

**Figure 10:**
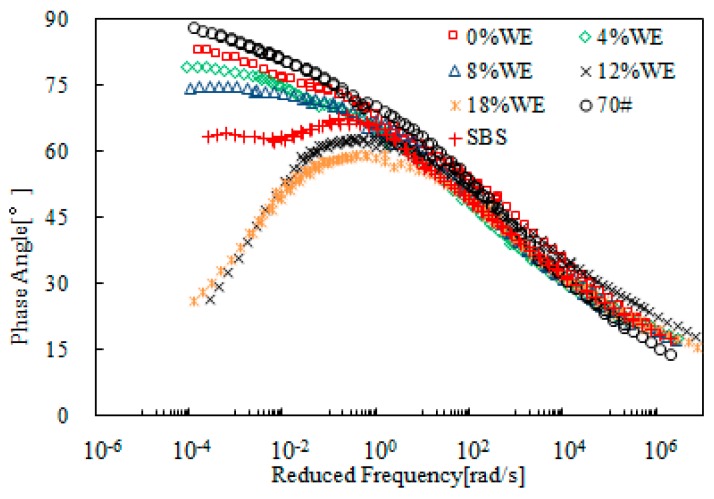
Master curves of the phase angle of WE-modified bitumen emulsion.

Figure 10 shows the master curves of phase angle of WE-modified bitumen emulsion. The effect of WE modification was relatively small for the phase angle at the high frequency region. Distinct change could be observed at the low frequency region, which corresponds to high temperature. The master curves of 4% and 8% WE-modified bitumen emulsion were located between that of the 70# base bitumen and SBS-modified bitumen. When compared with SBS-modified bitumen, 12% WE was needed to obtain a fundamental phase change. 

**Figure 11:**
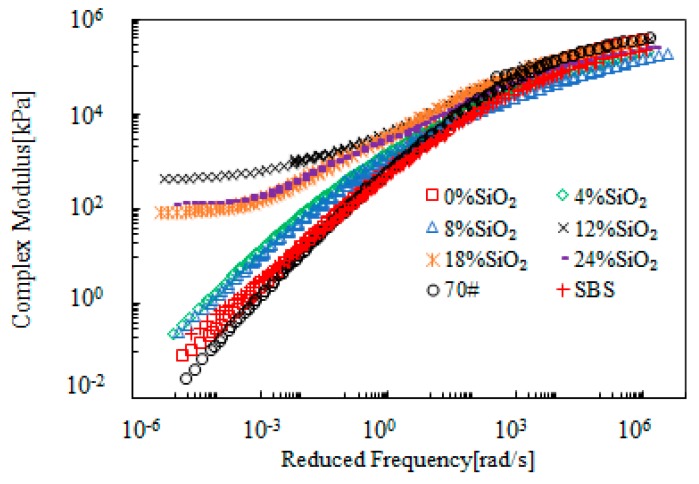
Master curves of the complex modulus of SiO_2_-modified bitumen emulsion.

**Figure 12:**
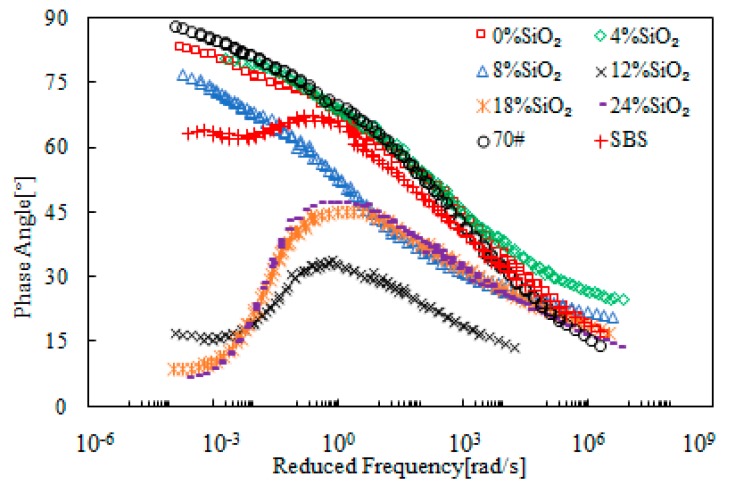
Master curves of the phase angle of SiO_2_-modified bitumen emulsion.

Figure 11 presents the master curves of the complex modulus of SiO_2_-modified bitumen emulsion. It shows that SiO_2_ had a more significant modification effect on the complex modulus at low frequency region when compared with the high frequency region. This indicated that SiO_2_ modification had a better improvement for high-temperature properties. When SiO_2_ dosage was below 8%, the change of the complex modulus was close to that of SBS-modified bitumen. A sudden increase on complex modulus at the low frequency region was observed when SiO_2_ dosage was beyond 12%. These observations are in agreement with the master curves of the phase angle presented in Figure 12. This strongly indicated a phase change which was beyond the traditional bitumen behavior. Therefore, SiO_2_ had a significant modification on bitumen emulsion and the critical dosage was 12%. 

**Figure 13:**
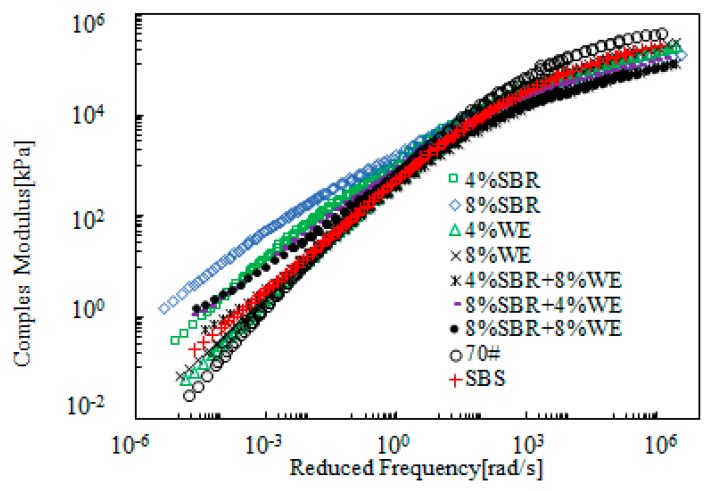
Master curves of complex modulus of SBR + WE-modified bitumen emulsion.

**Figure 14:**
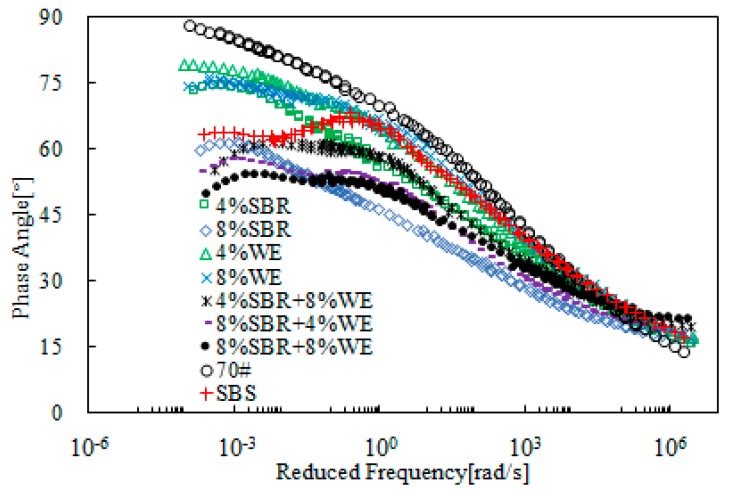
Master curves of the phase angle of SBR + WE-modified bitumen emulsion.

Figure 13 and Figure 14 present the rheological master curves of SBR + WE-modified bitumen emulsion. The benefit of SBR on further modification of WE-modified bitumen emulsion was clear. For instance, bitumen emulsion with 8% WE dosage only had a slight improvement on complex modulus, but addition of 4% SBR led to an obvious improvement. A similar phenomenon was observed with phase angle. Using single modification as a reference, it is clear that SBR was more predominant than WE on the improvement of rheological properties. Bitumen emulsion containing both SBR and WE had rheological properties superior to those of SBS-modified bitumen.

**Figure 15:**
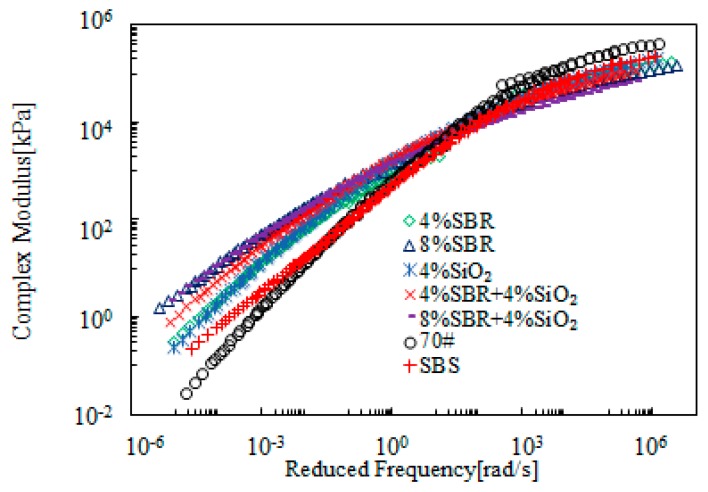
Master curves of complex modulus of SBR + SiO_2_-modified bitumen emulsion.

**Figure 16:**
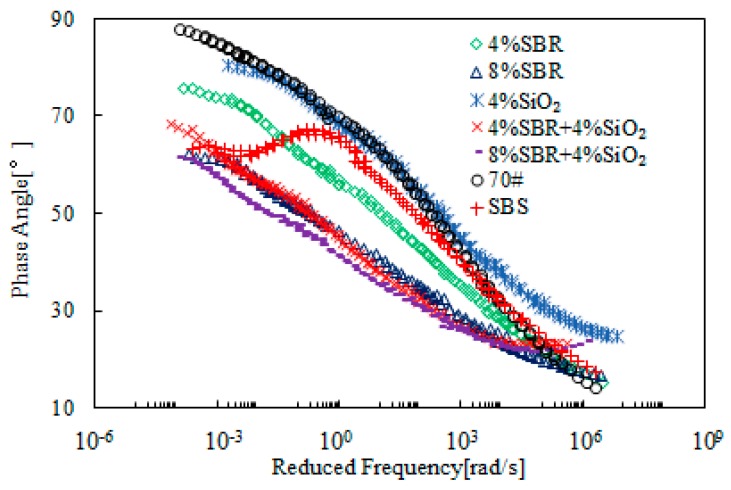
Master curves of phase angle of SBR + SiO_2_-modified emulsified asphalt.

Figure 15 and Figure 16 present the rheological master curves of SBR + SiO_2_-modified bitumen emulsion. In these figures, 4% SiO_2_ was added into bitumen emulsion modified with either 4% or 8% SBR and the effects of SiO_2_ was thus investigated. It was observed that there was a slight improvement on complex modulus at low frequency region. With respect to phase angle, bitumen emulsion containing 4% SBR was further improved while the emulsion with 8% SBR was hardly improved by the additional 4% SiO_2_.

**Figure 17:**
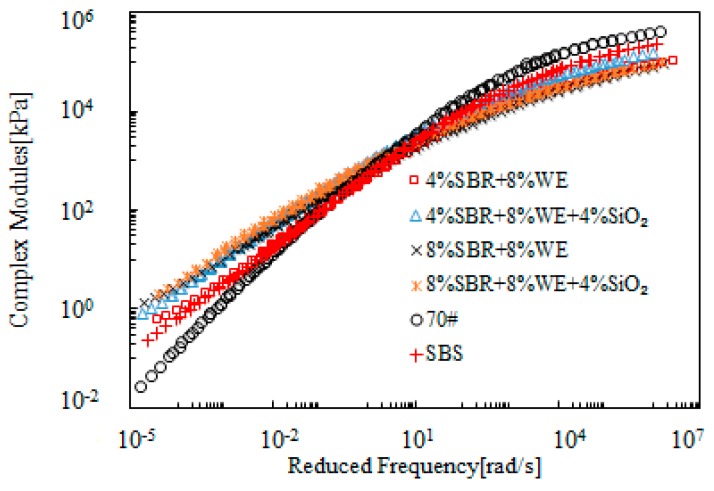
Master curves of complex modulus of SBR + WE + SiO_2_-modified bitumen emulsion.

**Figure 18:**
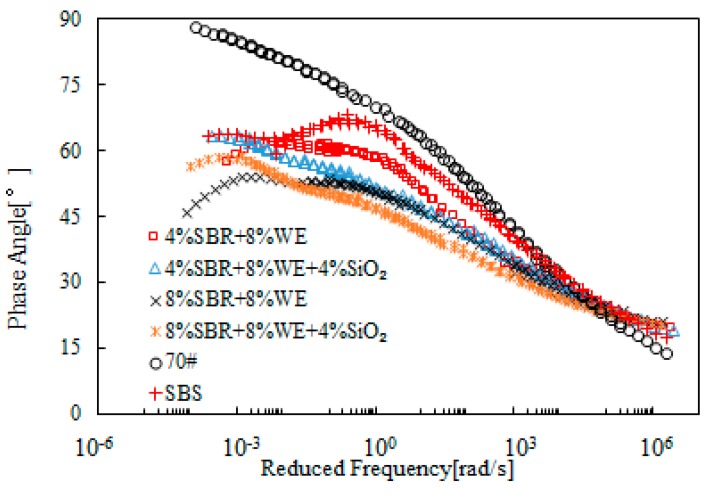
Master curves of phase of SBR + WE + SiO_2_-modified bitumen emulsion.

Figure 17 and Figure 18 present the master curves of complex modulus of SBR + WE + SiO_2_-modified bitumen emulsion. For the purpose of comparison, bitumen emulsion modified by the corresponding SBR + WE combination was included in these figures. SBR + WE + SiO_2_ further increased the complex modulus over a wide range of frequencies. This indicated a positive effect on high-temperature properties but a negative effect on low-temperature properties. Data obtained from phase angle indicated only a slight difference between SBR + WE and SBR + WE + SiO_2_-modified bitumen emulsion. At the low frequency region, bitumen emulsion containing two or three modifiers had rheological properties superior to those of SBS-modified bitumen, which indicated a good compatibility of the different modifiers in bitumen emulsion.

### 3.3. Relation between Bonding Strength and Rheological Properties

**Figure 19:**
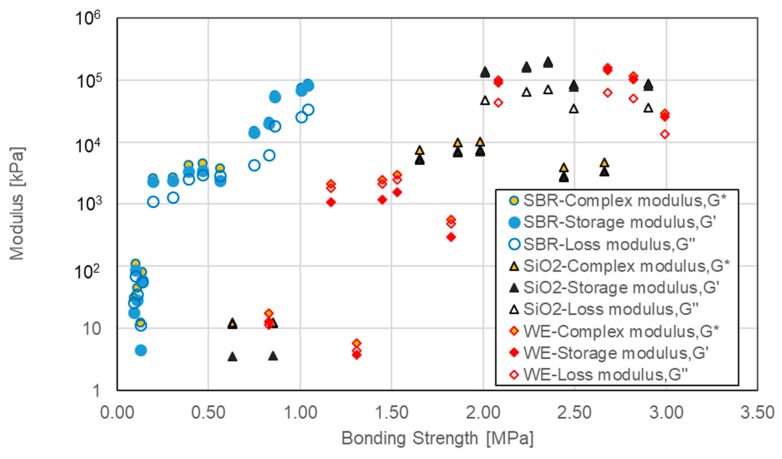
Relation between bonding strength and various modulus parameters.

Figure 19 shows the relation between bonding strength and various modulus parameters including complex modulus, storage modulus and loss modulus. Three types of individual modified bitumen emulsion including SBR, WE and SiO_2_ were involved. For each modified bitumen emulsion, the modifier dosage ranged from 0 to 24%. The general tendency seems to be same among complex modulus, storage modulus and loss modulus for a certain type of individual modified bitumen emulsion. Higher modulus tended to lead to higher bonding strength. This agreed with the results of single lap shear strength and storage modulus of ethylene vinyl acetate (EVA) copolymers reported by Park [24]. Park found the shear strength decreased with reduced storage modulus. These three types of modified bitumen emulsion exhibited distinct change trends. At a low level of modulus, below 100 kPa, WE-modified bitumen emulsion showed the largest bonding strength. At a medium level of modulus (10^3^–10^4^ kPa), SiO_2_-modified bitumen emulsion had the highest bonding strength. When the modulus was above 10^4^ kPa, WE-modified bitumen emulsion seemed to exhibit the largest bonding strength. For SBR-modified bitumen, the maximum bonding strength correlated with the largest modulus. However, the largest modulus of WE and SiO_2_-modified bitumen emulsion did not result in the highest bonding strength. This indicated that excessive stiffness and brittleness could result in decreased bonding strength. 

**Figure 20:**
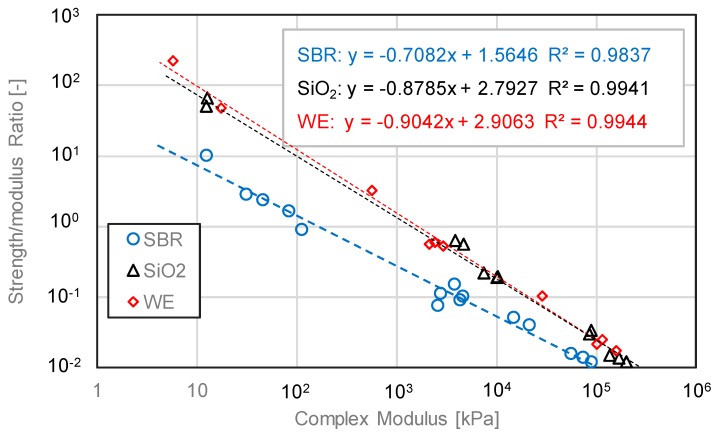
The measured ratio between bonding strength and complex shear modulus.

Figure 20 presents the measured ratio between bonding strength and complex shear modulus for various types of individual modified bitumen emulsion. It was observed that the strength/modulus ratio showed a linear relation with complex modulus in a log-log scale. The fitting results were shown in this figure with high coefficient of correlation. Lindner evaluated the adhesive strength and rheological properties of lightly crosslinked model acrylic networks and found that the stress level of the plateau after peak strength could be well predicted by complex modulus; the measured ratio between stress and modulus was 5 to 6 [25]. In our study, the strength/modulus ratio varied significantly ranging from 10^−2^ to 10^2^. A low modulus led to a high strength/modulus ratio. On the contrary, a high modulus resulted in low strength/modulus ratio. This trend indicated that bitumen strength increase might reach a standstill with increasing modulus or stiffness.

## 4. Conclusions

In this study, styrene butadiene rubber latex (SBR), waterborne epoxy adhesive (WE) and colloidal silica sol (SiO_2_) were used to prepare modified bitumen emulsion for cold mix asphalt applications. The effect of individual modifiers and combined modifiers on the bonding strength and rheological properties of modified bitumen emulsion were investigated. Based on the data obtained and the discussions presented, the following conclusions were made:

(1) The modifier dosage helped to balance the performance of modified bitumen emulsion in terms of improving its bonding strength without compromising its rheological properties. For a particular type of modifier, a less than sufficient dosage increased the bonding strength, but did not have a noticeable improvement of the rheological performance of the emulsion residue. Conversely, an over-dosage of modifier contributed to problems such as poor bonding strength, high stiffness, susceptible brittleness as well as high cost. Therefore, it is important to carefully select the type and dosage of modifier for a particular combination to optimize the performance of the bitumen emulsion.

(2) The addition of SBR, WE and SiO_2_ modifier noticeably improved the bonding strength of modified bitumen emulsion and the critical dosage at which the peak value occurred was 4%, 12% and 4% respectively. Among these modifiers, SiO_2_ modifier showed the most significant effect on bonding performance of modified bitumen emulsion. SBR-modified bitumen emulsion can be further modified using WE, SiO_2_ and a combination of both to obtain greater bonding strength.

(3) Modification of bitumen emulsion with combined SBR, WE and SiO_2_ modifiers improved its rheological performance. In particular, the modifiers increased the complex modulus and reduced the phase angle at the low frequency region. In general, effects on phase angle were more significant than those on the complex modulus, and thus could be used as a good indicator for modification effect.

(4) Modified bitumen emulsion indicated a phase change when modifier dosage was beyond 12%. This was indicated by the abrupt change in phase angle of its the rheological master curves when compared to that of SBS-modified bitumen. 

(5) Highly-modified bitumen emulsion can be prepared by using the combination of SBR, WE and SiO_2_. This method improved the rheological properties without compromising the bonding strength. For the ease of cold mix applications, WE and SiO_2_ can be added into the commonly used SBR-modified bitumen emulsion to prepare a compatible emulsion with improved performance.

(6) The bonding strength strongly depended on the rheological properties of modified bitumen emulsion. The measured ratio between bonding strength and complex shear modulus could vary ranging from 10^−2^ to 10^2^. 

This study gave a fundamental insight into the bonding and rheological properties of bitumen emulsion modified separately with SBR, WE, SiO_2_ as well as with the combinations of two or three of the modifiers. Highly-modified bitumen emulsion is expected to contribute to improved performance on moisture and rutting resistance for cold asphalt mix. This should be further validated by testing cold mixing asphalt in future work. 

## Figures and Tables

**Table 1:** Summary of modifier dosages for various types of modified bitumen emulsion.

Type of Modification	Type of Modifier	Dosage of Modifier
Individual Modification	SBR	4%, 8%, 12%, 18%, 24%
	WE	4%, 8 %, 12%, 18%, 24%
	SiO_2_	4%, 8%, 12%, 18%, 24%
Double Modification	SBR + WE	4% SBR + 8% WE
		8% SBR + 4% WE
		8% SBR + 8% WE
	SBR + SiO_2_	4% SBR + 4% SiO_2_
		8% SBR + 4% SiO_2_
Multiple Modification	SBR + WE + SiO_2_	4% SBR + 8% WE + 4% SiO_2_
		8% SBR +8% WE + 4% SiO_2_
